# Methodology and Experimental Protocol for Fatigue Analysis in Suggestopedia Teachers

**DOI:** 10.3390/brainsci14121215

**Published:** 2024-11-30

**Authors:** Gagandeep Kaur, Borislava Kostova, Paulina Tsvetkova, Anna Lekova

**Affiliations:** 1 Institute of Robotics, Bulgarian Academy of Sciences, Acad. Georgi Bonchev Str., 1113 Sofia, Bulgaria; borislava.kst@gmail.com (B.K.); p.tsvetkova.ir@gmail.com (P.T.); a.lekova@ir.bas.bg (A.L.); 2 Faculty of Information Sciences, University of Library Studies and Information Technologies, 1784 Sofia, Bulgaria

**Keywords:** electroencephalography (EEG), neurocognitive study, psychological assessment, fatigue, resting state EEG, suggestopedia, interrelations across EEG bands, theta band, alpha band, delta band, beta band

## Abstract

Background: Among all professions, teaching is significantly affected by psycho-social risks with approximately 33.33% of educators reporting work-related fatigue. Suggestopedia, an effective pedagogical approach developed in Bulgaria, claims to induce positive psychological and cognitive benefits in both teachers and students. In order to gather scientific evidence, given the above statement, we designed a methodology to detect fatigue in Suggestopedia teachers based on neurocognitive analysis and psychological assessment. Methods: An increase in the EEG theta and alpha band powers is considered among the most reliable markers of fatigue. The proposed methodology introduces a robust framework for fatigue analysis. Initially, the changes in EEG band powers using the resting state EEG activity before and after teaching are measured. Subsequently, validated psychological questionnaires are used to gain subjective feedback on fatigue. The study participants include a control group (traditional teachers) and the test group (suggestopedia teachers) to assess whether suggestopedia practice mitigates fatigue among teachers. Observations: In a pilot study, the EEG data was analyzed by evaluating the interrelations between EEG bands and the alpha–beta ratio. The results of the proposed study are expected to provide comprehensive analysis for the fatigue levels of teachers. In future research, our goal is to position the described methodology as a robust approach for evaluating cognitive and emotional states.

## 1. Introduction

### 1.1. Rationale

The European Trade Union Committee for Education (ETUCE) alerted in its 2023 report to the European Commission that the teaching profession is among the most affected by psycho-social risks [1]. Among the broader workforce, over 33.33% of the professionals reported overall work-related fatigue, and 27% reported depression and anxiety, worsened by their job. While this reduces the attractiveness of the profession and can potentially lead to retention issues in educational systems across Europe, the other concerning aspect of it is lower self-efficacy for teaching, lower job satisfaction and lower commitment. Research into the professional lives of teachers reveals specific challenges—a decrease in motivation, fatigue, and under-appreciation that adversely impacts their reward system and the demanding task of maintaining students’ attention [2,3,4,5].

At the same time, teachers and students practicing the Bulgarian pedagogical method of Suggestopedia experience positive affective states in class such as joy [6], happiness [7] and well-being in terms of the development of personality, creativity, talents [8], communicative and leadership skills [9], grace, love for fellow human beings, inspiration [9], refinement of character [10], and satisfaction in teaching because Suggestopedia is a synthesis of pedagogy, psychology and art [8,11,12], where teachers develop a multitude of skills in order to practice the method [7,13]. A positive effect on preventing and alleviating mental or emotional disorders is also observed [14].

Suggestopedia is one of the few pedagogical methodologies recommended by UNESCO as an exceptionally innovative and effective approach to teaching and learning [15,16]. It was developed and experimented with by a team of researchers and teachers under the leadership of the Bulgarian doctor and psychotherapist Prof. George Lozanov. Suggestopedia is an original pedagogical method for teaching and learning any educational subject. This method is known for its effectiveness in language learning, both for adults and children [8,17,18].

While the pedagogical framework of Suggestopedia offers a unique perspective on enhancing teacher satisfaction and mental health, it is crucial to understand the broader context of job stress that educators face, which significantly impacts their well-being and performance.

### 1.2. Job Stress and Well-Being Among Teachers

Job stress among teachers refers to the negative emotional experiences that educators face, often resulting in extreme mental and physical fatigue, heightened tension, frustration or distress. These feelings are typically triggered by factors such as long working hours, excessive workloads, and student misconduct [19]. In a 2005 study conducted on occupational stress, Johnson et al. reported that among twenty-six different studies monitoring psychological well-being, job satisfaction and physical health, teaching turned out to be among the top six most stressful professions [20]. A few other studies focused on the emotional regulation and mental well-being of teachers and also corroborated the occupational stress among teachers [21,22].

A recent review found that the widespread presence of chronic stress among teachers varied between 8.3% and 87.1%, while moderate to severe burnout affected 25.1% to 74.0% of teachers [23]. Factors influencing teachers’ stress and burnout include organizational and work-related aspects such as teaching experience, job satisfaction, the number of students in a class and the subjects taught, along with socio-demographic factors like gender, age and marital status [24]. High levels of occupational stress in teachers have been linked to numerous negative health effects, including fatigue, stress, burnout, anxiety and depression [25]. These issues can harm teachers’ job performance and productivity, indirectly affecting students. Chronic exposure to stress can result in burnout. The burnout syndrome is classified as a multilevel response syndrome resulting from prolonged stress and is characterized by physical and mental exhaustion, depersonalization, cynicism, a sense of helplessness and low self-efficacy [26]. Emotional exhaustion is frequently regarded as the core element of burnout and has been the focal point of numerous studies within the educational field [27,28,29].

On the other hand, studies also focus on factors that contribute to educators’ well-being and strategies to help them manage stress. Positive factors like job satisfaction and enthusiasm at work, along with the absence of mental or emotional stress, are essential components of teachers’ occupational well-being [30]. This concept relates to their optimal psychological functioning and experience in the workplace. A systematic review explores the factors impacting teachers’ occupational well-being, emphasizing the importance of organizational support and social-emotional competence in reducing stress [31]. Given the significant levels of stress and burnout among teachers and their detrimental effects, it is crucial to evaluate methods aimed at addressing these psychological challenges. Tackling these issues is essential for enhancing both teachers’ well-being and the educational system’s effectiveness.

A serious and ongoing deliberation among academic researchers focuses on teachers’ well-being, emphasizing the need to develop models that foster their emotional and social skills, which is evident in a recent book chapter on European demography [32]. Recently, platforms have been encouraging the dissemination of studies addressing teachers’ well-being, for instance, among secondary school teachers [33], pre-university teachers [34], and educational institutions across countries [35] and the probable mitigating measures. Interestingly, the Suggestopedia framework integrates these key elements, with the course curriculum reflecting core principles of benevolence, a promise of success for all and a strong focus on teacher training. This approach ensures that lessons are delivered in a highly motivational manner, enhancing the impact of the study material [36]. The Suggestopedia method and its relevance are described in the following section.

### 1.3. Suggestopedia Method and Fatigue

A recent 2019 study by Giuseppe Caruso [35] addressed the fatigue among teachers and eventual burnout in English language teaching and proposed the introduction of an emotion-focused pedagogical framework as a measure to mitigate it. The Suggestopedia method for language learning is already designed with a holistic framework that nurtures a balance between emotional well-being with the pursuit of delivering advanced conventional proficiency quickly [36]. Suggestopedia teachers, unlike their conventional counterparts, report absence of fatigue and more satisfaction in teaching using this method [37]. This perspective adds to why it is important to become familiar with Suggestopedia teaching methods in light of the challenges faced by the most valuable human resource in teaching: teachers.

The Suggestopedia lesson design is based on specific principles and tools existing permanently and simultaneously [38], aiming to achieve the optimal state for teaching and learning. The method uses visualization and imagination for classroom engagement, "concert reading" (reading with intonation and in rhythm with specifically selected classical music) to enhance concentration, comprehension and retention [18,38]. The teacher ensures the atmosphere is relaxed while the learning is imparted. The pedagogical approach of Prof. Lozanov is based on tender suggestion techniques such as music, art and role-play [39], which assist students in achieving a “positive mindset that learning is easy and fun” [6,40]. This approach maximizes practice on the subject by focusing on classroom interactions between students and minimizing the teacher’s presentation [41]. The success of the method is linked to delivering conversational proficiency three times more quickly than other methods for both adult and children students [41,42].

One of the crucial elements in achieving both outstanding learning results and teacher satisfaction in Suggestopedia is designing class activities to prevent fatigue from affecting either teachers or students during lessons [18,38]. According to prof. Lozanov, “if there is fatigue, there is no Suggestopedia” [38] and “relaxation is a prerequesite for quicker and easier memorization of the teaching matter” [6]. In the classroom, gentle suggestion techniques such as music, art and role-play [39] assist students in achieving a “positive mindset that learning is easy and fun” [40].

The relaxation that Suggestopedia seeks to achieve is not merely through various forms of passive behavior but primarily through thoughtfully designed class activities that aim to activate or inhibit specific brain structures, physiological functions and biochemical processes, as the effectiveness of learning depends on how knowledge is conveyed [14].

The instruction tools used in Suggestopedia and their effects on the well-being of students were extensively experimented within the Institute of Suggestopedia in Bulgaria between 1966 and 1991 with EGG, ECG and EMG technologies, psychological surveys, physiological examinations, observations and participant feedback [13,18,43]. Existing scientific research on Suggestopedia confirms that it does not register biomarkers of intensive mental work, and an increase in beta waves or reduction in alpha waves is absent [44]. Class activities calm the brain’s bioelectrical function and increase the capacity for memorization and learning [13,18].

The information from the review of existing scientific literature on the Suggestopedia method is summarized in Table 1. This table outlines the observed outcomes of the Suggestopedia method in terms of accelerated learning, activation of hidden cognitive reserves, reduced fatigue, and positive health changes. Each outcome is paired with corresponding psychological and physiological indicators. The methods used to investigate these outcomes include a range of qualitative and quantitative approaches, such as psychological and physiological surveys, EEG, ECG, and EMG recordings, as well as questionnaires and medical examinations. It illustrates the research methodology and the observed effects of the method on students. There is a shared observation in the Suggestopedia community that the positive effects of the methodology also transfer to teachers, as they are part of an interactive system [43], but future research needs to confirm this hypothesis with scientific methods.

The proposed methodology extends the scientific research on neurocognition initiated by the Institute of Suggestopedia utilizing a combination of EEG, ECG, EMG data, psychological surveys, physiological examination and qualitative observations [43]. This methodology is structured within the following framework:

1. Using resting state EEG activity, which is a novel element that overcomes the restrictions of movements of participants.
2. Combining EEG-based analysis with validated psychological questionnaires, as described in Section 1.4 and Section 2.3, which is in line with the holistic propositions made by Balevsky in his 1973 study [43].
3. Another novelty in the proposed research is the shift of focus entirely onto the teachers.

We aim to find empirical evidence to substantiate fatigue levels and the stimulation of the reward system in teachers. EEG is a reliable measure for detecting fatigue, which is relevant in the present context and has been validated in numerous empirical studies using brain oscillations: delta (up to 4 Hz), theta (4−8 Hz), alpha (8−13 Hz), and beta (13−30 Hz) frequencies. The following section highlights the adequacy of the EEG technique for investigating fatigue.

### 1.4. EEG Neuroimaging for Fatigue

The neurocognitive approach in our study refers to using changes in the neuronal activity to study mental fatigue. In most cases, mental-fatigue is studied in controlled laboratory settings, and changes in EEG band powers, such as the increase in frontal theta and alpha, are commonly relied upon. Prior studies on the detection and assessment of fatigue and alertness levels have used the EEG technique and facial feature detection systems [45,46,47,48,49,50,51,52]. In [46], a team of researchers from Manipal University, led by Sharma, explored advanced methods for detecting drowsiness and fatigue, using image processing for face and eye movement detection algorithms. The study proposes a three-phase model that utilizes the Viola–Jones algorithm to identify facial features, detect yawning and track faces. Yawn detection and eye tracking are achieved using template matching and correlation coefficients. Features from these phases are combined to produce a binary result, classifying fatigue states. The authors reported that when fatigue surpasses a specific threshold, an alarm is activated, indicating a high level of accuracy. The authors of another study [48] explored the advancement of classification algorithms used for detecting fatigue through EEG in recent years, focusing on two types of algorithms: intra-subject and cross-subject. They observed a gradual increase in research utilizing deep learning and transfer learning and emphasized the fact that researchers must create effective experimental paradigms and gather valid standard data to advance fatigue detection methods or systems for broader application and greater accuracy. The authors in [49], evaluated four types of EEG activities—delta (δ), theta (θ), alpha (α), and beta (β)—during a monotonous driving session involving 52 participants (36 males and 16 females). The results indicated consistent delta and theta activities over time, a slight reduction in alpha activity, and a notable decrease in beta activity (*p* < 0.05). All four algorithms demonstrated an increase in the ratio of slow-wave to fast-wave EEG activities as time progressed. These findings have important implications for fatigue detection. The authors of [50] propose a drowsiness detection system using physiological signals that decompose EEG signals into wavelet sub-bands to reveal more detailed information beyond the raw signals. They extracted and combined nonlinear features from EEG sub-bands, integrating information from EEG and eyelid movements and utilizing efficient machine learning for classification. This approach demonstrated high detection accuracy and very fast computation speed. The proposed algorithm has the potential to be developed into monitoring and warning systems to help prevent mental fatigue that reduces work efficiency in various settings such as driving, aviation and education.

Our investigation takes a novel approach by testing fatigue levels based on a real classroom teaching experience. Another innovation is the use of resting state EEG activity, which addresses a major challenge in EEG-related studies—the mitigation of movement-related artifacts. The changes in resting state EEG activity prior to teaching are compared against resting state EEG activity after the teaching to detect persistent markers of fatigue. We determine its persistence using changes in alertness levels. In the context of teaching, the minimal effect of fatigue would be a decline in the quality of the performance.

Additionally, the inter-relationships between frequency bands are considered, and therefore, the measure of relative power across delta, theta, alpha, and beta bands are studied to obtain a well-rounded analysis of alertness levels in the context of persistent cognitive fatigue. The significance of an inter-relational approach during the examination of cognitive engagements is emphasized in a 2013 study by Thalia Harmony in [53]. The present study examines these alertness levels using the EEG beta band activity (13–30 Hz), which is known to signify alertness levels [54] and is often used as a key marker for both motor imagery and behaviors. As we examine the alertness levels, we also assume that the beta activity should not be evaluated independently but relative to the alpha activity. This is because the alpha activity increases and beta activity decreases as alertness levels go down and fatigue sets in.

Earlier EEG-based studies have been conducted for fatigue detection where the participant was subjected to tasks of various complexities. The parietal alpha activity power (Pz) and the amplitude of the P300 wave have been used as markers of fatigue, and theta power at Fz was tested for different levels of workload [55]. In the context of the present study, the detection of mental fatigue related to teaching is significant as it can lead to potential burnout and negatively affect a teacher’s motivation [56]. On a physical level, fatigue is often characterized by an overwhelming sensation of tiredness, while on a neurocognitive level fatigue is marked by decreased alertness [57]. The repercussions of fatigue on performance are also discussed by Kathner et al.’s 2014 study [55], wherein time and frequency domain features were simultaneously employed for the determination of fatigue. Furthermore, if the engagement is not rewarding, then it can lead to a lack of motivation. Studies have reported that fatigue hinders both performance capacity and motivation [58,59].

### 1.5. The Aim of the Study

The aim of the study is to evaluate the EEG activity and psychological feedback data for the detection of a teacher’s fatigue. We assume that changes in resting state EEG activity before and after the session act as reliable bio-markers for analyzing teacher fatigue. We specifically studied the relationships between EEG band powers and the alpha–beta ratio to establish reliable conclusions regarding teacher fatigue. Our underlying assumption is that multiple perspectives will lead to a dependable assessment of fatigue. Therefore, a comprehensive analysis of various EEG bands along with the alpha–beta ratio is essential. The proposed methodology aims to evaluate teachers’ brain activity by monitoring the changes in the resting-state EEG recorded before and after teaching.

The results presented in this paper are based on a pilot study conducted with a participant engaged in Suggestopedia teaching methodology. An increase in theta band power is considered the most reliable bio-marker of fatigue. At the end of this study, a reduction in theta band power was observed. Furthermore, an increase in the alpha band power was observed. This is in alignment with the framework of the Suggestopedia class that is designed to induce a relaxed state of mind. Our ongoing study seeks to further explore and validate our methodology of using changes in the resting state EEG activity before and after teaching.

This research employs a systematic approach to explore the interplay between teaching methods and cognitive states, specifically focusing on fatigue and motivation in Suggestopedia teachers. Central to this methodology is the examination of teachers’ brain activity through resting-state EEG recordings taken before and after the teaching sessions. By analyzing changes in EEG activity as potential biomarkers for mental fatigue, we aim to uncover insights into how teaching impacts cognitive states.

**Figure 1:**
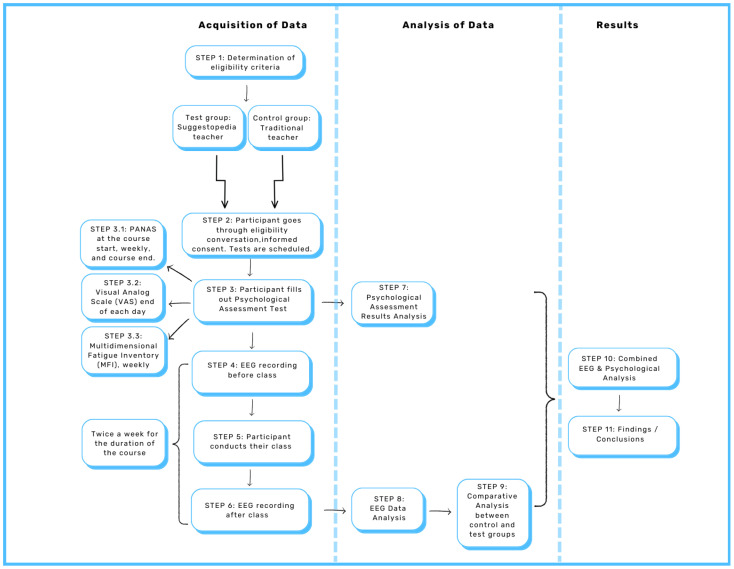
Flowchart of EEG and psychological data analysis for evaluating fatigue in Suggestopedia teachers.

The dual EEG assessments, alongside psychological evaluations, allow for a comprehensive analysis of fatigue and motivation. Specifically, we investigate the interrelations between EEG band powers and the alpha–beta ratio to draw informed conclusions about teachers’ fatigue. This multifaceted approach ensures that our findings are robust and reliable, ultimately enhancing our understanding of the effects of Suggestopedia teaching on educators’ cognitive well-being. These aspects are presented in Figure 1 and enumerated in the following steps. In order to assess the specific effects of Suggestopedia classes on EEG metrics, a control condition is introduced. The control group consists of the same number of teachers practicing a traditional pedagogical approach. Control groups play a crucial role in maintaining the internal validity of research.

STEP 1: Inclusion and exclusion criteria for the test group (Suggestopedia) and the control group (traditional teaching) are established to ensure initial equivalence:
  (a) Teachers in both groups must practice the respective standardized teaching methods, confirmed either by a diploma or workplace assurance (for Suggestopedia, adherence to the classical method is required);
  (b) Participants must have comparable years of teaching experience; and
  (c) Participants must teach similar subjects.
STEP 2: The research team conducts conversations with each teacher (hereafter referred as participant) from the control and experimental group ensuring that they meet the eligibility criteria, listed in Step 1, before taking their EEG recording. The research participant is then informed about the study’s ethical guidelines, objectives, procedures, and potential risks before providing consent. The participant fills out a consent form, and a timeline for data collection is established.
STEP 3: The participant fills out psychological assessment tests.
  STEP 3.1: PANAS at the course start, weekly, and at the course end. The PANAS is a valuable tool for psychologists who want to monitor shifts in clients’ positive and negative emotions on a weekly basis. Its sensitivity to short-term changes in affect makes it suitable for tracking not only the immediate impact of therapy sessions or interventions but also the results of different activities like teaching.
  STEP 3.2: Multidimensional Fatigue Inventory (MFI) administered on a weekly basis. This questionnaire is a 20-item self-report instrument designed to measure fatigue. It covers the following dimensions: general fatigue, physical fatigue, mental fatigue, reduced motivation and reduced activity. This provides frequent feedback on a teacher’s fatigue levels during the course of teaching. In addition to the MFI, all participants are required to rate their fatigue using a Visual Analog Scale (VAS), where 0 represents “very alert” and 10 signifies “extremely fatigued ”.
STEP 4: The participant undergoes EEG recordings done pre-class. A comparison between the resting state EEG activity before and after class teaching is used to draw inferences on the fatigue and motivation levels of the teacher. At this stage, the resting state EEG activity of the teacher is recorded prior to the class, which serves as a baseline.
STEP 5: The participant conducts their usual teaching activities. A Suggestopedia class consists of specific activities that the teacher is trained to perform. These activities are designed to induce a state of relaxed awareness in students, maximizing the effectiveness of the class while minimizing fatigue. Key techniques include:
  (1) Reinforcing positive suggestions and avoiding negative conditioning.
  (2) Presenting a large amount of study material to stimulate curiosity.
  (3) Vocabulary harmonized in unison with psychological and artistic means.
  (4) Reading and listening with intonation and in rhythm with classical music.
  (5) Role-play.
  (6) Involvement of curated music and arts in the environment and role-play activities.
  (7) Purposefully timed changes between activities and breaks.
  The learning activities used in the traditional classrooms differ significantly to those included in the Suggestopedia classes. Contrary to Suggestopedia, traditional teaching is mainly teacher-centered and focuses on lecturing, demonstrations, note-taking, homework, question and answer sessions and testing, repetition-based activities, copying exercises, and memorization of certain parts of the study material, such as vocabulary.
STEP 6: The participant then has EEG recordings performed post-class. The resting state EEG activity of the teacher is recorded after the class. This serves as a comparison with the pre-class EEG.
  Steps 3, 4, and 5 are executed twice a week for the duration of the course. To balance between avoiding participant exhaustion and gathering sufficient data, we decided to collect data twice a week. The course consists of different stages, each varying in intensity for the teacher. This design ensures that data samples are collected for each stage.
STEP 7: Analysis of psychological assessment results using descriptive and comparative statistics.
STEP 8: EEG data analysis for each of the groups. Interrelations between EEG bands (delta, theta, alpha, beta) and the alpha–beta ratio are analyzed to draw conclusions related to fatigue.
STEP 9: Comparative analysis between the control and test groups.
STEP 10: The final inferences regarding fatigue within groups and between groups are drawn using the neurocognitive and psychological data from steps 6 and 7.
STEP 11: Findings/conclusions.

## 2. Materials and Methods

### 2.1. Participation

Participation in this study is open to traditional teachers (control group) and teachers practicing classical Suggestopedia (test-group) of any gender. The participants are invited through in-person seminars and visits where the study objectives are explained. If these interactions evoke interest among teachers, they may choose to participate voluntarily and are free to withdraw from the study at any stage.

### 2.2. Ethics

The study adhered to ethical guidelines, ensuring information about the study’s objectives, procedures, risks and benefits before providing consent. Confidentiality was assured and participants’ identities were anonymized in the reporting of results. This research received approval with protocol No. 1/2023 from the ECSR-IRBAS (Ethics Committee for Scientific Research, Institute of Robotics, Bulgarian Academy of Science).

### 2.3. Questionnaires

The Positive and Negative Affect Schedule (PANAS) test [60], used to measure positive and negative emotional states, is administered to participants at the beginning of the study to establish a baseline. Subsequently, the PANAS test is provided at weekly intervals until the study’s conclusion. Furthermore, the Multidimensional Fatigue Inventory (MFI) [61]) is administered at the end of each week. In addition to the MFI, all participants are required to rate their fatigue using a Visual Analog Scale (VAS), where 0 represents “very alert” and 10 signifies “extremely fatigued”. Information on the time-frame for the study is provided in Table 2.

### 2.4. EEG Device

The Mitsar-smartBCI device with 19 EEG channels and gel-based electrodes mounted in an elastic fabric cap following the 10–20 system of the American Electroencephalographic Society [62] was selected for the EEG recording. The corresponding electrode positions are Fp1, Fp2, F7, F8, Fz, F3, F4, C3, Cz, C4, T3, T4, T5, T6, P3, Pz, P4, O1 and O2. A universal reference electrode ‘ref’ placed after Fz along the central mid-line is built into the device as the common-reference electrode. Additionally, the electrodes A1 and A2 were placed in the mastoid positions, but these were not used as references in our EEG recording montages. The ground electrode was placed at AFz. EEG data were sampled at 256 Hz, as a singular option. Data collection was controlled by the WinEEG software package, version 2.134.107(04.2019), designed for Mitsar-EEG systems. Data processing, storage and online display were conducted on a laptop (Intel Core i5 2.5 GHz, 16 GB RAM, Microsoft Windows 11 Professional 64-bit). These details are tabulated in Table 3.

### 2.5. Study Design

As per the study aims and proposal, a combined psychological and EEG-based (neurocognitive) analysis of fatigue will be conducted. Initially, the Positive and Negative Affect Schedule (PANAS) will be administered, and a resting state EEG will be recorded before the beginning of course classes to establish baseline data.

Throughout the course, the PANAS, along with a fatigue monitoring test, will be administered weekly. Additionally, the resting state EEG will be recorded twice weekly, both before and after class, with each session lasting 3 min. These recordings will be carried out for the entire duration of the course, which is expected to last 4–5 weeks. The EEG recording and the psychological tests will be mainly recorded in the corresponding classrooms where participants actually conduct classes. This is a deliberate decision to ensure that an ambient teaching environment is captured without the restrictions posed by a controlled laboratory environment. Subsequently, the data processing and documentation will be mainly conducted at the Institute of Robotics, Bulgarian Academy of Sciences.

As a test of the neurocognitive aspect of methodology, a pilot study was conducted with one participant over a period of 2–3 weeks in an actual Suggestopedia teaching setting. A total of five sets of recordings were acquired, with each set consisting of one recording taken before and one after the class. Four of the recordings were used for further analysis, while one set was discarded due to an inability to stabilize the EEG data baseline, despite multiple precautions. This decision was made to ensure the integrity of the data.

A stage-2 study, with additional participants from the control group and test group, is planned for future research. The control group will provide a baseline for comparison with the test group—the Suggestopedia teachers. Both groups will be treated identically; see Figure 1. In this way, the approach will ensure that any differences observed between the two groups at the study’s conclusion can be confidently attributed to the methodology used by the educators—the Suggestopedia method and the traditional method of teaching.

### 2.6. Procedure

The participant will sit in a comfortable chair with armrests, with the Mitsar EEG system mounted on their scalp. They are instructed not to engage in active thought processes, avoid physical movements and focus on a black screen with a white cross at the center to control gaze movements. Resting state EEG data will be recorded for a duration of 3 min before the class. Subsequently, another set of EEG data will be recorded for 3 min after the class. EEG data will be further processed for the analysis of fatigue levels associated with Suggestopedia teaching.

### 2.7. Neurocognitive Approach

#### 2.7.1. EEG Data Processing

The data analysis will be performed using the EEGlab toolbox integrated within the Matlab environment. The data pre-processing has the following steps:

- The channel locations and event markers to mark the beginning and end of the resting-state trial will be integrated into the channel data.
- Data epochs of 15 s each will be extracted from the pre-teaching class and post-teaching EEG recordings. The raw EEG data will be a two-dimensional array (channel* time-samples).
- Next, the basic finite impulse response (FIR) filter option will be used from the EEGlab menu to bandpass the data between 2 and 30 Hz. The FIR filters do not distort the signal phase or cause infinite oscillations, which make them suitable for filtering the sensitive EEG epoch. This will be performed to remove low-frequency artifacts as well as preserve the signals between (2–30) Hz for further analysis.
- The noisy channels will be interpolated to minimize data loss. The interpolation will be performed within the EEGlab environment by selecting the channels to be interpolated. A spherical interpolation method will be used for channel interpolation.

The decision to analyze and select the following five electrode positions: the frontal (Fz), central (Cz), posterior (Pz), and occipital (O1 and O2), is based on the following considerations [52]:

- Frontal electrode position (Fz): This position was selected due to the relevance to the interactive nature of Suggestopedia teaching, which involves continuous decision-making and association of frontal theta activity with fatigue.
- Motor area and the Cz electrode position: This is included because the Suggestopedia teaching involves calculated physical movements during rearrangements of the classroom, concert sessions and role-playing.
- Parietal electrode position, Pz: The ability to identify and interpret sensory information to understand and respond to stimuli involves the parietal region, which works in synergy with the frontal areas of the brain to process incoming sensory stimuli. An examination of the changes in this position before and after teaching is crucial for studying fatigue.
- Occipital Area (O1 and O2): We include the occipital positions to monitor the changes in visual attention before and after the class, with the inquiry of fatigue. O1 and O2 are used instead of Oz due to device limitations.

#### 2.7.2. Computing the Measures of Power

In the proposed study, we focus on calculating the absolute and relative powers of the EEG signals. The powers will be computed using the Darbeliai-EEGlab toolbox within the MATLAB environment. This toolbox provides an interface for performing these calculations wherein the users provide input parameters such as data-epochs, sampling rate, channel locations, and frequency bands of interest. The toolbox automates the power calculation and subsequent steps for calculating the absolute and relative power values. The darbeliai toolbox within EEGlab is used to ensure that the EEG data are processed and analyzed consistently, as it is among the well-established practices in EEG research.

#### 2.7.3. Statistical Analysis

In terms of the psychological data, including the PANAS (Positive and Negative Affect Schedule) and the Multidimensional Fatigue Inventory (MFI), descriptive statistical analyses will be performed for the control and test groups, traditional teachers and suggestopedia teachers, respectively. The data will be summarized using means, standard deviations and percentages, providing insights into the overall trends and distributions of emotional states and fatigue levels across the study participants. Comparative statistical analysis will be conducted between the test and control groups.

Similarly, the EEG signal band power (delta, theta, alpha, and beta bands) will also undergo descriptive statistical analysis. This will involve calculating the mean, standard deviation, and variance of the band power across different time points (pre- and post-teaching sessions). These statistical measures will help to characterize any changes in neural activity, allowing for comparisons between the recorded sessions.

## 3. Expected Results

We expect that the proposed methodology will provide valuable insights for determining the fatigue levels of teachers following the traditional pedagogy (control group) and Suggestopedia framework (test group). The combination of psychological assessments and neurocognitive measures in the framework described in Section 2.5 ensures a robust methodology. The variations in EEG data recorded before and after the teaching are expected to cause fluctuations in band powers in addition to the changes in self-reported psychological tests. The expected evaluation is described in the following sections:

### 3.1. Psychological Evaluation of Fatigue

Due to the inherent positive reinforcement methods used in Suggestopedia, the teacher’s emotional states, measured by PANAS, are expected to show a pattern of stability. Fatigue levels measured by MFI are expected to fluctuate based on different stages of the course. There may be differences between the more intensive phases of teaching compared to the less intensive phases. The psychological evaluation, administered weekly, over the entire duration of our proposed study makes for a comprehensive assessment of fatigue.

### 3.2. Neurocognitive Evaluation of Fatigue

It is anticipated that teachers’ EEG recordings will show differences in the band power before and after the class. A shift is expected in the theta and alpha bands, as studies have identified these bands as reliable biomarkers of fatigue. The changes in delta band power will be evaluated across participants to determine the relationship between delta band power and fatigue. Changes in beta band power are also expected, as this band is associated with alertness. The alpha–beta ratio will be monitored to verify teacher fatigue.

**Figure 2:**
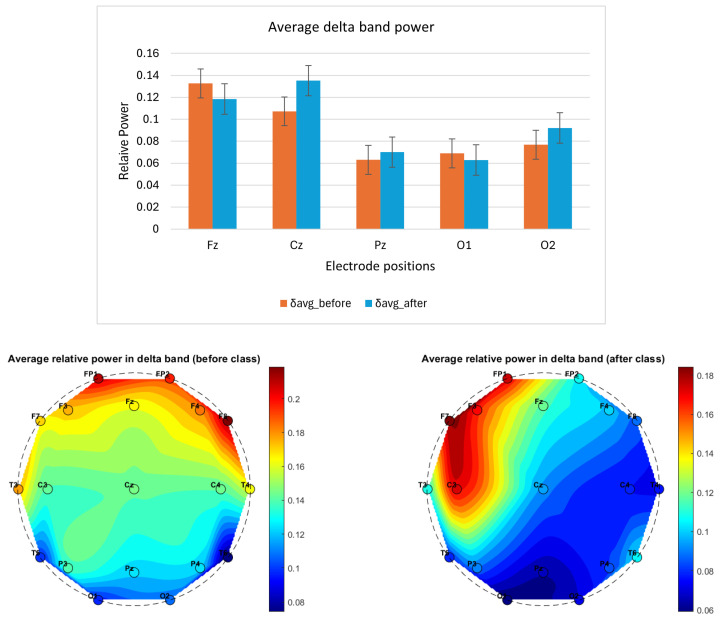
The column plot (top row) shows EEG band power variation before (orange) and after (blue) a Suggestopedia class in the delta (2–4)Hz band. Each column depicts the average power for data recorded over the days. The mid-line (Fz, Cz, Pz) and occipital electrode (O1 and O2) positions are selected for the study as per the description given in Section 2.7.1. The *x*-axis specifies the electrode position and the *y*-axis represents relative power. The topographical maps (bottom row) show the average relative power in delta band, before and after the class for all 19 channels.

**Figure 3:**
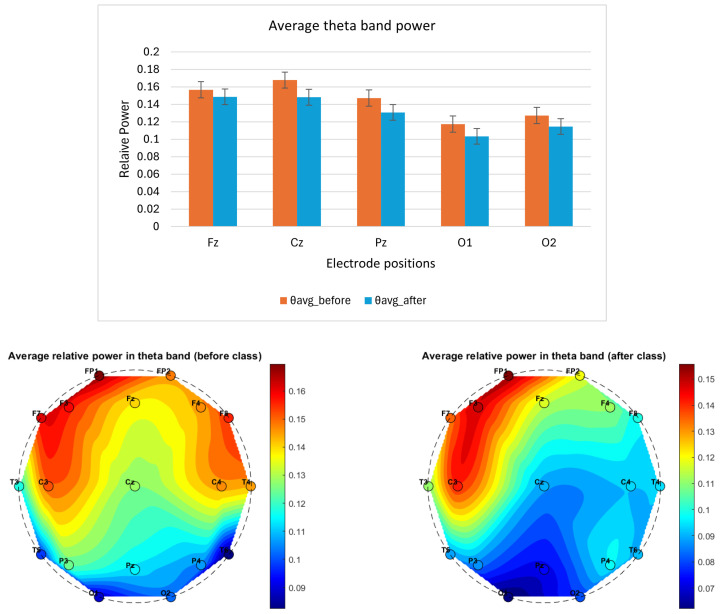
The column plot (top row) shows EEG band power variation before (orange) and after (blue) a Suggestopedia class for theta (4–8) Hz band. Each column depicts the average power for data recorded over all of the days. The mid-line (Fz, Cz, Pz) and occipital electrode (O1 and O2) positions are selected for the study as per the description given in Section 2.7.1. The *x*-axis specifies the electrode position, and the *y*-axis represents relative power. The topographical maps (bottom row) show the average relative power in theta band before and after the class for all 19 channels.

**Figure 4:**
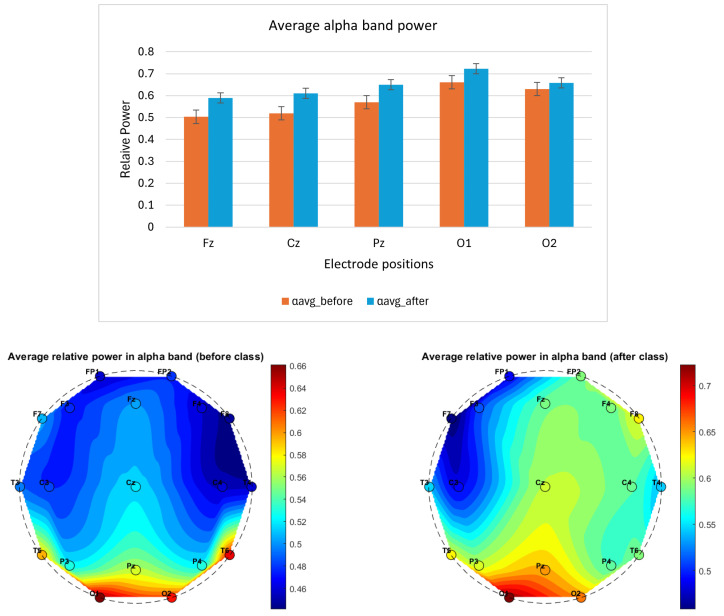
The column plot (top row) shows EEG band power variation before (orange) and after (blue) a Suggestopedia class for alpha (8–13) Hz band. Each column plot depicts the average power for data recorded over all of the days. The mid-line (Fz, Cz, Pz) and occipital electrode (O1 and O2) positions are selected for the study as per the description given in Section 2.7.1. The *x*-axis specifies the electrode position, and the *y*-axis represents relative power. The topographical maps (bottom row) show the average relative power in the alpha band, before and after the class for all 19 channels.

**Figure 5:**
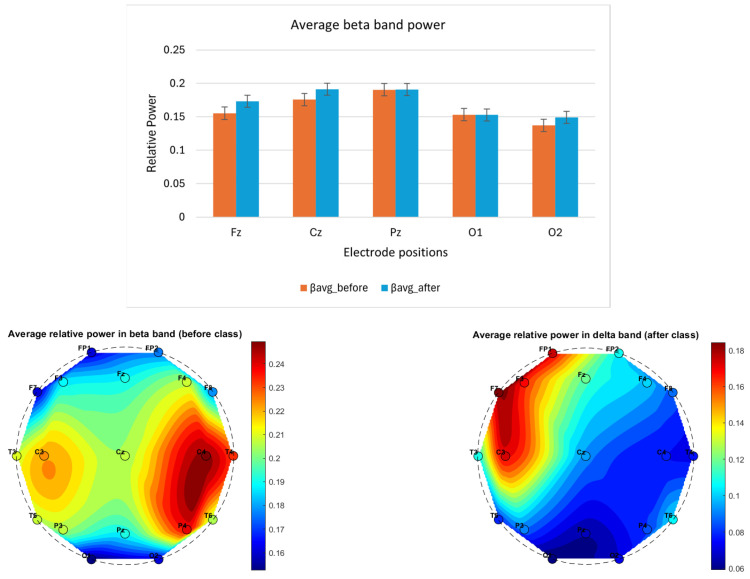
The column plot (top row) shows EEG band power variation before (orange) and after (blue) a Suggestopedia class for beta (13–30) Hz band. Each column depicts the average power for data recorded over all of the days. The mid-line (Fz, Cz, Pz) and occipital electrode (O1 and O2) positions are selected for the study as per the description given in Section 2.7.1. The *x*-axis specifies the electrode position, and the *y*-axis represents relative power. The topographical maps (bottom row) show the average relative power in the beta band before and after the class for all 19 channels.

**Figure 6:**
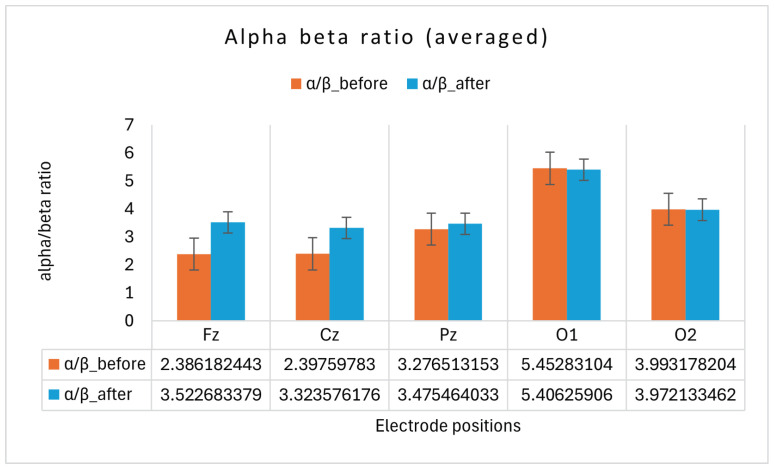
The ratio between the alpha (8–13 Hz) and beta (13–30 Hz) frequency bands, known as the alpha–beta ratio, before (orange) and after (blue) the teaching session is illustrated. The *x*-axis represents electrode positions, and the *y*-axis represents the averaged alpha–beta ratio obtained over the entire session for all of the participants.

A pilot neurocognitive study was conducted on a single participant over a 3-week period, with EEG data recorded before and after a 3 h Suggestopedia class. Although the sample size was limited, important patterns emerged from averaging four days of EEG recordings. Notably:

- The average delta power exhibited a variable pattern across electrode positions, suggesting the need for further investigation to clarify its correlation with fatigue, as shown in Figure 2.
- The average theta power across all the selected electrode positions (Fz, Cz, Pz, O1 and O2) was lower after class compared to before, as shown in Figure 3. The topographical distribution of theta activity (bottom row Figure 3) indicated a decrease from before teaching to after teaching. This was observed across the 19 electrode positions. Through empirical observation, at the frontal and central positions, the theta power reduced more for the left-hemispheric (FP1, F3, F7, C3) compared to the right. The hemispheric dominance will be studied with a larger number of participants.
- The average alpha band power was higher after class, as shown in Figure 4. The topographical distribution of alpha activity indicated an increase from before teaching to after teaching. This was observed across the 19 electrode positions. The hemispheric dominance will be studied with a larger number of participants.
- The average relative power for the beta band was higher for the mid-line and occipital positions, as shown in Figure 5 (top row). For a wider representation, the topographical map for each of the 19 electrode positions is shown in the bottom row of Figure 5. The significance of this observation will be confirmed with a higher number of participants.
- The average alpha–beta ratio was observed to be higher at central mid-line positions (Fz, Cz, Pz) and slightly lower at occipital sites (O1, O2) after a class, as shown in Figure 6.

These findings, while inconclusive because of the small sample size, provide a foundation for conducting a larger study due to the presence of observable patterns. These patterns highlight the value of evaluating the interrelations between multiple EEG bands.

## 4. Discussion

In this paper, a protocol to examine the fatigue and motivation of participants, using neurocognitive and psychological indicators, is presented. The motivation behind this investigation is derived from the numerous reports of negative cognitive effects of occupational stress, such as fatigue, lowered motivation, and anxiety. The Suggestopedia method, on the other hand, is designed in a way that it bypasses fatigue and induces relaxation. This contrast led to the study design, which includes both EEG techniques and psychological feedback to examine fatigue in teachers.

### 4.1. Neurocognitive Approach

The neurocognitive evaluation of fatigue, by recording the resting state EEG activity of the participants in their own environment, prior to and after teaching is one of the novel approaches in our method that overcomes the limitation of a controlled and simulated laboratory environment. Neurocognitive analysis will be carried out using the interrelations between EEG bands. Specifically, heightened theta activity at the frontal, central and posterior sites is referred to as a robust biomarker of fatigue [52]. Interrelations between EEG bands emerge when other EEG bands are simultaneously observed for fatigue detection, assuming that they will converge toward a common inference. In this direction, the alpha activity will be evaluated, an increase in which the fatigue studies almost unanimously associate with fatigue. Research indicates that increased alpha activity can be a marker of a relaxed, alert mind, as mentioned in a 2007 study by Knyazev [63]. It is essential to consider the context and specific goals of Suggestopedia, which promotes a structured teaching and learning environment designed to prevent fatigue and promote alertness.

As a continuation of the inter-relational analysis, an examination of beta power will also be carried out. At occipital positions, beta activity signifies active visual engagement, and at parietal positions, it signifies active processing of sensory information. However, if the brain loses the capacity to perform and slows down, in an attempt to maintain alertness levels, an increase in the beta activity is the brain’s way of compromising without affecting the performance, which is especially relevant to the frontal sites. The delta waves have traditionally been associated with the non-rapid eye movement (nREM) sleep stage that propagates across the cortex. However, there are also data that support an increase in delta power in states relevant to motivation [63], and arithmetic tasks [64]. Another study [65] by Harmony et al. monitored EEG activity during a mathematical task and observed an increase in delta power in the pre-frontal cortex and an increase in both delta and beta powers in the left-parietal cortex.

Thus, the proposed study to examine interrelations between EEG bands in the context of a teacher’s classroom engagements is a significant approach. The comparative analysis between traditional and Suggestopedia teaching is included in order to assess the specific effects of Suggestopedia classes on EEG metrics of fatigue.

### 4.2. Psychological Approach

The psychological approach, employing validated questionnaires [60,61], provides subjective insight into participants’ cognitive states regarding fatigue and motivation. This complements the neurocognitive approach achieved through EEG analysis. A general questionnaire assessing the teacher’s positive and negative emotions is administered at both the beginning and end of the study. In addition, a more specific questionnaire assessing fatigue levels is administered following EEG recording sessions at the end of each week throughout the study. The interrelations between EEG bands, the alpha–beta ratio, and psychological tests facilitate a comprehensive analysis of the data, aiming to corroborate participants’ feedback with interpretations of brain activity.

### 4.3. Future Work and Limitations

As the next step of the proposed methodology, the study will be conducted over a larger set of participants. Thus, we will verify the methodology by empirical evidence and statistical analysis. In future research, our goal is to expand the described methodology as a robust approach for evaluating broader cognitive and emotional states.

At the same time, we expected the following limitations stemming from this holistic approach:

- The weekly psychological assessment tests for subjective feedback can also introduce underlying individual biases in self-reporting. This may introduce variability, making it challenging to correlate with the EEG data.
- There is a temporal mismatch between the EEG data recording and post-session questionnaires. Their influence can only be known at the end of the study.

Given the challenges posed by using both EEG analysis outcomes and psychological questionnaires for detecting fatigue levels, this remains a subject for future studies.

## Figures and Tables

**Table 1:** Summary of observed outcomes in Suggestopedia studies.

Observed Outcomes	Observed Indicators	Type of Investigation
Accelerated learning	Conversational proficiency three times more quickly than other methods for both adult and children students [14,41,42].	Questionnaires, Observations.
Activating hidden reserves	Hypermnesia, provoked hyper creativity: improved memory, positive emotional states such as joy [6,40], love for fellow human beings and grace [9], inspiration and creativity, personal development [8], and leadership skills [10].	Psychological and physiological surveys and observations.
Lack of fatigue	Despite effective educational outcomes, biomarkers of intensive mental work are not registered (increase in beta waves or reduction in alpha waves is absent) [44]. Class activities calm the brain’s bioelectrical function and increase the capacity for memorization and learning [13,18].	Psychological, physiological surveys and observations, EEG, ECG, EMG, questionnaires, and medical examinations.
Positive change in the state of health	No disturbances in the emotional state, the nervous system, or the sleep-wake cycle [14]. Positive psychoprophylactic and psychotherapeutic effects.	Psychological and physiological surveys and observations, EEG, ECG, EMG, questionnaires, and medical examinations.

**Table 2:** Time-frame for psychological and fatigue assessments during the study.

Time-Point	Test Administered	Details
Beginning of the study	PANAS-test (Positive and Negative Affect Schedule)	Baseline questionnaire
After class	Visual Analog Scale (VAS)	Administered at the end of each class which is tested.
Weekly intervals	Multidimensional Fatigue Inventory (MFI) and PANAS-test	Administered at the end of each week
End of study	Final PANAS test and Fatigue questionnaires (MFI, VAS)	End-of-study emotional and fatigue assessment

**Table 3:** EEG recording setup overview.

Component	Details
Device Used	Mitsar-smartBCI device
Electrodes and Cap	Gel-based electrodes mounted in an elastic fabric cap following the 10–20 system
Number of EEG Channels	19 EEG channels
Electrode Placement	Fp1, Fp2, F7, F8, Fz, F3, F4, C3, Cz, C4, T3, T4, T5, T6, P3, Pz, P4, O1, O2
Reference Electrode	Universal reference electrode placed after Fz along the central mid-line
Additional Reference Electrodes	A1 and A2 (placed at mastoid positions, but not used in recording montages)
Ground Electrode	AFz
Sampling Rate	256 Hz (singular option)
Software for Data Collection	Win-EEG software package
Data Processing System	Laptop (Intel Core i5 2.5 GHz, 16 GB RAM, Windows 11 Professional 64-bit)

## Data Availability

The data used to support the findings of this study are available (in anonymized form) upon request submitted to Gagandeep Kaur (gdk@ir.bas.bg or gdk.irbas@gmail.com). The data is not publicly available at this stage because the present paper represents a study protocol for an ongoing study.

## Abbreviations

The following abbreviations are used in this manuscript:

ETUCE	European Trade Union Committee for Education
EEG	Electroencephalograph
UNESCO	United Nations Educational, Scientific and Cultural Organization

## References

[1] Flanagan L., Cordelier O., Keller A., Kerperien T., Lange D., Strukelj B., Flocken S., Donegan J. (2023). ETUCE Position on a European Comprehensive Approach to Mental Health.

[2] Kalsoom T., Akhter M., Mujahid A.H., Saeed A., Kausar M. (2017). Teachers’ perception regarding effect of reward system on teachers’ performance at elementary level. Bull. Educ. Res..

[3] Ormiston H.E., Nygaard M.A., Apgar S. (2022). A Systematic Review of Secondary Traumatic Stress and Compassion Fatigue in Teachers. School Mental Health.

[4] Kumari J., Kumar J. (2023). Influence of motivation on teachers’ job performance. Humanit. Soc. Sci. Commun..

[5] Asl E.S., Asl N.S., Asl A.S. (2014). The Erosion of EFL Teachers’ Content and Pedagogical-Content Knowledge Throughout the Years of Teaching Experience. Procedia-Soc. Behav. Sci..

[6] Beer F. (1978). Report on the School Experiment “Suggestopedia in Elementary School”. J. Class. Suggest..

[7] Dumitrana M. (2007). Sugestopaedia as a Stimulating Teaching Strategy.

[8] Gateva E. Development of the potential creative talents of the personality through Suggestopedia. Proceedings of the International Conference on Suggestopedia.

[9] Bodurova V. (2021). Grace in Suggestopedia or Are We Ready for the Legacy of Doctor Georgi Lozanov?. J. Class. Suggest..

[10] Peev I.P., Todorova-Hlavacik B.A. Possibilities for the Application of Suggestopedia for Forming and Developing Communicative Skills and Leadership Culture in the Senior Naval Staff as a Factor for the Navigation Safety in the 21 Century (Innovations in the Sea Education of the Future). Proceedings of the International Scientific Conference “Science and Technology for Sustainable Maritime Development”.

[11] Jane W., Bancroft W.J. (1976). Suggestology and Suggestopedia: The Theory of the Lozanov Method.

[12] (2017). Vihrovenia. What Is Suggestopedia. https://foundation-vihrovenia.bg/what-is-suggestopedia.

[13] Balevsky P. (1973). EEG changes during the process of memorization under normal and suggestive conditions, Problems of Suggestology. Proceedings of the International Symposium of the Problems of Suggestology.

[14] Lozanov G. (1987). On Some Problems of the Anatomy, Physiology and Biochemistry and Brain Activity in the Global—Artistic Approach in the Modern Suggestopedic Training. Suggestology and Personality Development.

[15] Lozanov G. (1978). Suggestology and Suggestopedia—Theory and Practice.

[16] Schinckel P.M. (2015). Teaching methodology: An overview of desuggestive learning and accelerated learning. J. Soc. Humanit..

[17] Montagner D. (2022). Suggestopedia Meaning and Message in the 21st Century. https://fastlearningschool.com/blog/suggestopedia-meaning-and-message-in-the-21st-century.

[18] Lozanov G. (2005). Suggestopedia, Desuggestive Learning: A Communicative Method of the Hidden Reserves Within Us.

[19] Roeser R., Schonert-Reichl K.A., Jha A., Cullen M., Wallace L., Wilensky R., Oberle E., Thomson K., Taylor C., Harrison J. (2013). Mindfulness training and reductions in teacher stress and burnout: Results from two randomized, waitlist-control field trials. J. Educ. Psychol..

[20] Johnson S., Cooper C., Cartwright S., Donald I., Taylor P., Millet C. (2005). The experience of work–related stress across occupations. J. Manag. Psychol..

[21] Herman K.C., Reinke W.M., Eddy C.L. (2020). Advances in understanding and intervening in teacher stress and coping: The Coping-Competence-Context Theory. J. Sch. Psychol..

[22] Greenier V., Derakhshan A., Fathi J. (2021). Emotion regulation and psychological well-being in teacher work engagement: A case of British and Iranian English language teachers. System.

[23] Chang H. (2022). Stress and Burnout in EFL Teachers: The Mediator Role of Self-Efficacy. Front. Psychol..

[24] Hadi A.A., Naing N.N., Daud A., Nordin R.B., Sulong M.R. (2009). Prevalence and factors associated with stress among secondary school teachers in Kota Bharu, Kelantan, Malaysia. Southeast Asian J. Trop. Med. Public Health.

[25] Agyapong B., Obuobi-Donkor G., Burback L., Wei Y. (2022). Stress, Burnout, Anxiety and Depression Among Teachers: A Scoping Review. Int. J. Environ. Res. Public Health.

[26] Zysberg L., Orenshtein C., Gimmon E., Robinson R. (2017). Emotional intelligence, personality, stress, and burnout among educators. Int. J. Stress Manag..

[27] Goetz T., Becker E.S., Bieg M., Keller M.M., Frenzel A.C., Hall N.C. (2015). The Glass Half Empty: How Emotional Exhaustion Affects the State-Trait Discrepancy in Self-Reports of Teaching Emotions. PLoS ONE.

[28] Arens A.K., Morin A.J.S. (2016). Relations Between Teachers’ Emotional Exhaustion and Students’ Educational Outcomes. J. Educ. Psychol..

[29] Taxer J.L., Frenzel A.C. (2015). Facets of teachers’ emotional lives: A quantitative investigation of teachers’ genuine, faked, and hidden emotions. Teach. Teach. Educ..

[30] Diener E., Suh E.M., Lucas R.E., Smith H. (1999). Subjective Well-Being: Three Decades of Progress. Psychol. Bull..

[31] Viac C., Fraser P. (2020). Teachers’ well-being: A framework for data collection and analysis. Proceedings of the OECD Education Working Papers.

[32] Martinsone B., Therese M.J., Wiesner C., Angelika K.A.Z., Martinsone B., Therese M.J., Wiesner C., Angelika K.A.Z. (2024). Wellbeing in the teachers’ profession: Theoretical Considerations and Multi-Cultural Research in Europe. Teachers’ Professional Wellbeing—A Digital Game Based Social-Emotional Learning Intervention.

[33] Pagán-Castaño E., Sánchez-García J., Garrigos-Simon F.J., Guijarro-García M. (2021). The Influence of Management on Teacher Well-Being and the Development of Sustainable Schools. Sustainability.

[34] Manasia L., Pârvan A., Macovei M. (2020). Towards a Model of Teacher Well-Being from a Positive Emotions Perspective. Eur. J. Investig. Heal. Psychol. Educ..

[35] Caruso G. (2019). Facing EL teachers’ burnout through motivation. J. Pedagog. Res..

[36] Ivanova G., Dimova-severinova D. (2021). The Role of Happiness in Applying Suggestopedia and Fostering the Language Learning Process The Role of Happiness in Applying Suggestopedia and Fostering the Language Learning. J. Soc. Stud. Educ. Res..

[37] Karagiozova R., Roerich P.O.F. (2021). The Heritage of Prof. Georgi Lozanov, Phd, in the cultural domain of Bulgarian Schools. J. Class. Suggest..

[38] Lozanov G. (1975). The Essence, History, and Experimental Perspectives of the Suggestopedic System in Foreign Language Learning. Suggest. Suggest..

[39] Feliciano Bonilla G. (2015). An Adaptation of Suggestopedia: Enhancing ESL Learners’ Motivation Through Music, Relaxation Techniques, and Role Playing. Master’s Thesis.

[40] Ragil K., Susanto M. (2014). Suggestopedia method in the teaching and learning process. J. Mhs. Teknol. Pendidik..

[41] Astutik Y. (2019). The effect of using suggestopedia among students’ speaking ability. Engl. Lang. Focus ELIF.

[42] Cisneros Castillo A.A. (2023). Suggestopedia Activities as a Teaching Strategy to Develop Reading Comprehension in Eighth-Year Students at Academia Militar San Diego–Ibarra. Bachelor’s Thesis.

[43] Balevsky P. (1973). Telemetric Registration of the Functional State of the Teacher-Student System in the Course of the the Suggestopedic Process of Instruction. Problems of Suggestology. Proceedings of the International Symposium of the Problems of Suggestology.

[44] Bancroft W.J. (1997). Suggestopedia, biofeedback and the search for the alpha state. J. Acc. Learn. Teach..

[45] Rezaee Q., Delrobaei M., Giveki A., Dayarian N., Haghighi S.J. Driver Drowsiness Detection with Commercial EEG Headsets. Proceedings of the 2022 10th RSI International Conference on Robotics and Mechatronics (ICRoM).

[46] Sharma V.P., Yadav J.S., Sharma V. (2022). Deep convolutional network based real time fatigue detection and drowsiness alertness system. Int. J. Electr. Comput. Eng. IJECE.

[47] Othmani A., Sabri A.Q.M., Aslan S., Chaieb F., Rameh H., Alfred R., Cohen D. (2023). EEG-based neural networks approaches for fatigue and drowsiness detection: A survey. Neurocomputing.

[48] Wang F., Wan Y., Li M., Huang H., Li L., Hou X., Pan J., Wen Z., Li J. (2023). Recent Advances in Fatigue Detection Algorithm Based on EEG. Intell. Autom. Soft Comput..

[49] Jap B.T., Lal S., Fischer P., Bekiaris E. (2009). Using EEG spectral components to assess algorithms for detecting fatigue. Expert Syst. Appl..

[50] Chen L., Zhao Y., Zhang J., Zou J.-Z. (2015). Automatic detection of alertness/drowsiness from physiological signals using wavelet-based nonlinear features and machine learning. Expert Syst. Appl..

[51] Lawhern V., Kerick S., Robbins K.A. (2013). Detecting alpha spindle events in EEG time series using adaptive autoregressive models. BMC Neurosci..

[52] Tran Y., Craig A., Craig R., Chai R., Nguyen H. (2020). The influence of mental fatigue on brain activity: Evidence from a systematic review with meta-analyses. Psychophysiology.

[53] Harmony T. (2013). The functional significance of delta oscillations in cognitive processing. Front. Integr. Neurosci..

[54] Gola M., Kamiński J., Brzezicka A., Wróbel A. (2012). Beta band oscillations as a correlate of alertness—Changes in aging. Int. J. Psychophysiol..

[55] Käthner I., Wriessnegger S.C., Müller-Putz G.R., Kübler A., Halder S. (2014). Effects of mental workload and fatigue on the P300, alpha and theta band power during operation of an ERP (P300) brain-computer interface. Biol. Psychol..

[56] Skaalvik E.M., Skaalvik S. (2021). Theory and practice Teacher burnout: Relations between dimensions of burnout, perceived school context, job satisfaction and motivation for teaching. A longitudinal study. Teach. Teach..

[57] Gordon P.C., Zrenner C., Desideri D., Belardinelli P., Zrenner B., Brunoni A.R., Ziemann U. (2018). Modulation of cortical responses by transcranial direct current stimulation of dorsolateral prefrontal cortex: A resting-state EEG and TMS-EEG study. Brain Stimul..

[58] Craig A., Tran Y., Wijesuriya N., Boord P. (2006). A controlled investigation into the psychological determinants of fatigue. Biol. Psychol..

[59] Craig A., Tran Y., Wijesuriya N., Middleton J. (2012). Fatigue and tiredness in people with spinal cord injury. J. Psychosom. Res..

[60] Watson D.B., Clark L.A., Tellegen A. (1988). Development and validation of brief measures of positive and negative affect: The PANAS scales. J. Personal. Soc. Psychol..

[61] Smets E.M.A., Garssen B., Bonke B., de Haes J. (1995). The Multidimensional Fatigue Inventory (MFI) psychometric qualities of an instrument to assess fatigue. J. Psychosom. Res..

[62] Mitsar. EEG Device. https://mitsar-eeg.com/.

[63] Knyazev G.G. (2007). Motivation, emotion, and their inhibitory control mirrored in brain oscillations. Neurosci. Biobehav. Rev..

[64] Dimitriadis S.I., Laskaris N.A., Tsirka V., Vourkas M., Micheloyannis S. (2010). What does delta band tell us about cognitive processes: A mental calculation study. Neurosci. Lett..

[65] Harmony T., Fernández T., Silva J., Bosch J., Valdés P., Fernández-Bouzas A., Galán L., Aubert E., Rodríguez D. (1999). Do specific EEG frequencies indicate different processes during mental calculation?. Neurosci. Lett..

